# In Vitro Study of Surface Changes Induced on Enamel and Cementum by Different Scaling and Polishing Techniques

**DOI:** 10.3290/j.ohpd.b927695

**Published:** 2021-01-28

**Authors:** Behrouz Arefnia, Martin Koller, Gernot Wimmer, Adrian Lussi, Michael Haas

**Affiliations:** a Dentist, Department of Dental Medicine and Oral Health, Medical University Graz, Graz, Austria. Wrote the manuscript.; b Dentist, Department of Dental Medicine and Oral Health, Medical University Graz, Graz, Austria. Performed in vitro tests.; c Dentist, Department of Dental Medicine and Oral Health, Medical University Graz, Graz, Austria. Contributed substantially to discussion.; d Professor, Department of Operative Dentistry and Periodontology, Center for Dental Medicine, University of Freiburg, Freiburg, Germany. Proofread the manuscript.; e Professor, Department of Dental Medicine and Oral Health, Medical University Graz, Graz, Austria. Idea.

**Keywords:** cementum, enamel, hand instruments, substance loss, surface roughness, ultrasonic air polishing

## Abstract

**Purpose:**

To determine how the currently available techniques of scaling and root planing, used either alone or with additional polishing techniques, affect the substance thickness and surface roughness of enamel and cementum.

**Materials and Methods:**

After extraction, impacted third molars were prepared and subjected to air polishing with a nonabrasive powder, ultrasonic scaling, or hand instrumentation. All three techniques were performed alone and in combinations for a total of 9 treatment groups. The control group consisted of untreated surfaces. Optical microcoordination measurements were conducted to separately assess substance loss, mean roughness depth (Rz), and roughness average (Ra) on enamel and cementum. The Rz results were analysed using a t-test for paired samples.

**Results:**

Air polishing alone and with additional rubber-cup polishing using a paste were the only two approaches which caused no enamel loss. Both groups also entailed less cementum loss (≤ 20 µm) than any of the other seven groups, and both yielded the most favorable Rz results on enamel. Air polishing alone was the only group to reveal no significant change in Rz from untreated cementum (p = 0.999). The other 8 approaches statistically significantly reduced the surface roughness of cementum (p ≤ 0.017).

**Conclusion:**

Air polishing with a nonabrasive powder yielded the best hard-tissue preservation. Combining any of the scaling techniques with additional polishing was not beneficial; on the contrary, they caused even more abrasion of hard tissue on both enamel and cementum.

Periodontal disease is an inflammatory disease caused by a bacterial infection of the tooth-supporting tissues.^17^ The prevalence of periodontitis in adults is over 46%^6^ and is a major dental public-health problem.^7^ Periodontitis is associated with a dysbiosis of the oral microbiome.^12^ The oral cavity provides various habitats for microbial colonisation;^5^ to date, more than 770 prokaryotic taxa which colonize the oral cavity and the human aerodigestive tract have been described in the literature thanks to newly developed sequencing methods.^8^ The direct effect of dysbiotic oral microbiota on systemic diseases is not fully understood, but good oral hygiene and professional maintenance using different techniques and instruments is stated to be important to prevent bacterial dissemination to other parts of the body.^10^

The mid-1980s saw a fundamental change in the treatment of periodontitis, following the demonstration that subgingival scaling and root planing was as effective as open flap debridement.^15^ This was a revolutionary finding at the time, eliciting a paradigm shift in periodontal treatment concepts that has since paved the way toward optimising periodontal tissue preservation. Subgingival debridement is today an integral part of any nonsurgical treatment of periodontal disease and, in addition to these first-line causal applications, is also used during surgery and supportive periodontal therapy.^14^

Any instrumentation of tooth surfaces for biofilm removal will cause some amount of substance loss. Loss of cementum should ideally remain confined to the 3–7 µm layer of endotoxin invasion in addition to the biofilm. The thickness of the cementum layer is known to vary with root areas, tooth types, and patient age. At the cervical level, it was reported to range in an age-dependent fashion (11−20 compared to 51−76 years) from 54 ± 2 up to 128 ± 4 µm.^24^ Generally speaking, the thickness of cementum is considered to be 250 µm. It is apparent from these figures that any excessive instrumentation can quickly eliminate the cementum layer and initiate defect healing.

Traditional methods of root planing are documented to cause losses in root substance of 6.3–55.9 µm with ultrasonic scalers, 93 µm with sonic scalers, and over 100 µm with curettes or rotary instruments.^1,11,22^ The recent development of nonabrasive powders based on glycine, erythritol combined with chlorhexidine, or trehalose has led to a revival of air polishing.^9,19,20^ Compared to ultrasonic scaling, this approach is similarly effective in removing biofilm, offers an exposure time of only 5 s per addressed surface of enamel or cement, causes no significant loss of cementum substance even after prolonged application,^23^ and has been shown to match the clinical parameters of ultrasonic scaling at least in single-rooted teeth.^13^

Besides the damage that may be inflicted on the root surface, another crucial factor for periodontal healing is the surface roughness achieved by root planing. Bühler et al^3^ reported roughness values of up to 7 µm after air polishing with nonabrasive powders, which is comparable to untreated root surfaces, and confirmed the clinical validity of this finding in a systematic review of 17 studies.^2^ Camboni and Donnet^4^ presented similar results on human enamel, reporting flawless cleaning with erythritol plus chlorhexidine and discussing the usefulness of additionally applying a polishing paste.

We designed this in vitro study (i) to determine how all major techniques of scaling and root planing affect the surface roughness and substance thickness of enamel and cementum, and (ii) to assess the respective surface qualities after additional polishing.

## Materials and Methods

### Specimen Preparation

**Fig 1:**
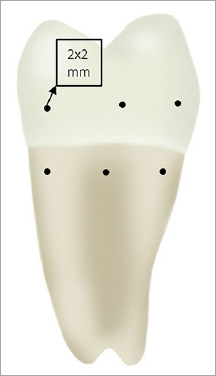
Schematic illustration of the analyzed sections.

Five impacted third molars were used for this study following their surgical removal. Using a diamond bur, punctiform markings were made as reference points to obtain processing fields 2 x 2 mm in dimension on both the enamel and root surfaces of each tooth (Fig 1). This totalled five untreated reference surfaces and 45 treatment surfaces; five for each treatment were available for analysis. Then the teeth were stored in sterile saline for a maximum of 3 weeks. During this time, they were processed and analysed using various techniques of debridement both with and without additional polishing.

### Experimental and Control Groups

All procedures were carried out by an experienced dentist (MK). A digital letter scale was used for continuous intraexaminer calibration of the force applied by the instruments (ultrasonic, hand, and contra-angle handpiece) to the surfaces in Newtons (N) to ensure that not too much pressure was applied. To maintain the working distance of 2 mm with the air-polishing device, a distance holder was attached to it using a self-curing, acrylic die material (GC Pattern Resin LS, GC; Tokyo, Japan) and a metal pin. Table 1 lists these treatment groups, including the scaling procedures and pertinent details on each of the techniques and devices. The following materials were used:

**Table 1:** Overview of the various techniques evaluated both alone and with additional rubber-cup polishing (using a paste) and/or air polishing (using a nonabrasive powder)

Group	Technique(s)	Device	Time/surface	Pressure/force	Movement
1A	Air polishing	EMS Airflow Master	5 s/2 mm distance	1.8 bar, 45°	Wiping
1B	Air polishing	EMS Airflow Master	5 s/2 mm distance	1.8 bar, 45°	Wiping
	Rubber-cup polishing	Contra-angle handpiece	60 s/surface/paste	0.3 N	Circular
2A	Ultrasonic	EMS Piezon Master	5 s/surface	0.3 N	Brushstroke
2B	Ultrasonic	EMS Piezon Master	5 s/surface	0.3 N	Brushstroke
	Air polishing	EMS Airflow Master	5 s/2mm distance	1.8 bar, 45°	Wiping
2C	Ultrasonic	EMS Piezon Master	5 s/surface	0.3 N	Brushstroke
	Rubber-cup polishing	Contra-angle handpiece	60 s/surface/paste	0.3 N	Circular
3A	Hand instrumentation	Scaler/curette	One stroke per surface	0.3 N	Scaling
3B	Hand instrumentation	Scaler/curette	One stroke per surface	0.3 N	Scaling
	Air polishing	EMS Airflow Master	5 s/2mm distance	1.8 bar, 45°	Wiping
3C	Hand instrumentation	Scaler/curette	One stroke per surface	0.3 N	Scaling
	Rubber-cup polishing	Contra-angle handpiece	60 s/surface/paste	0.3 N	Circular
3D	Hand instrumentation	Scaler/curette	One stroke per surface	0.3 N	Scaling
	Air polishing	EMS Airflow Master	5 s/2mm distance	1.8 bar, 45°	Wiping
	Rubber-cup polishing	Contra-angle handpiece	60 s/surface/paste	0.3 N	Circular

For the materials used with each device, see Materials and Methods section. All techniques were applied under standardised conditions.

- Air polishing: EMS Airflow Master with a nonabrasive polishing powder of 7-µm particle size (Perio Plus; EMS Electro Medical Systems; Munich, Germany)
- Ultrasonic scaling: the widely-used Piezon Master (EMS Electro Medical Systems) with an appropriate tip (PS Perio Slim, EMS Electro Medical Systems)
- Hand instrumentation: scaler (M23 Universal Scaler, Deppeler; Rolle, Switzerland) and curette (13/14 Rigid Gracey Curette, Hu-Friedy; Chicago, IL, USA).
- Rubber-cup polishing: contra-angle handpiece with a Hawe Prophy cup (Kerr; Orange, CA, USA) and polishing paste RDA 170 > 120 > 40 (ProphyCare, Directa; Upplands Väsby, Sweden) followed by RDA 7 (Proxyt, Ivoclar Vivadent; Schaan, Liechtenstein).

After every procedure, the surfaces were thoroughly rinsed for 30 s using sterile saline solution (NaCl 0.9%, Fresenius Kabi Austria; Graz, Austria). In addition, a control group of untreated enamel and untreated cementum was included for comparison of the various scaling and additional polishing approaches.

### Evaluation of Surface Roughness and Substance Loss

We performed these comparisons as required by the DIN 4768 German standard for surface roughness, assessing parameters of both surface roughness and substance loss with an optical microcoordination measuring unit (InfiniteFocus G5, Alicona Imaging; Graz, Austria). This is a noncontact optical system that works on a three-dimensional measuring principle based on focus variation, with a resolution of down to 10 nm.

The parameters of mean roughness depth (Rz) and roughness average (Ra) were obtained for defined measuring sections. Rz is calculated from the maximum peak-to-valley distances (i.e. sums of highest peak and deepest valley) of these sections (Fig 2a), and Ra represents the mean profile deviation from the center line (Fig 2b).

**Fig 2a:**
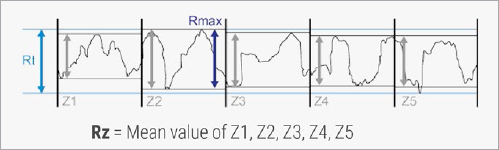
Illustration of the outcome parameter Rz (mean roughness depth). Rz represents the average peak-to-valley height of the roughness profile and is calculated from the maximum peak-to-valley heights (Rmax) of consecutive sampling lengths (courtesy of Alicona Imaging).

**Fig 2b:**
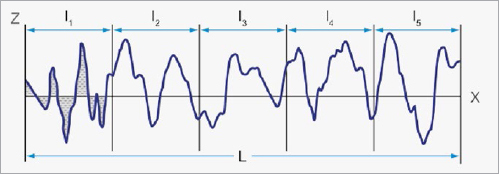
Illustration of the outcome parameter Ra (roughness average). Ra is the arithmetic average of the absolute values of the roughness profile ordinates. An example is given by the shaded area, which is divided by the sampling length (l1) (courtesy of Alicona Imaging).

Five surfaces per treatment group were processed and the mean values calculated.

### Statistical Analysis

For better presentation and clarity of the surface changes induced by each debridement technique, we based the statistical evaluations on the average peak-to-valley distance of the roughness profile (Rz). These per-technique results, also including the untreated control group of enamel and cementum, were subdivided to reflect the arithmetic means for enamel and cementum separately. One-way ANOVA followed by Bonferroni’s post-hoc comparisons were performed in all statistical analyses using SPSS (version 25.0; IBM, Armonk, NY).

## Results

**Fig 3:**
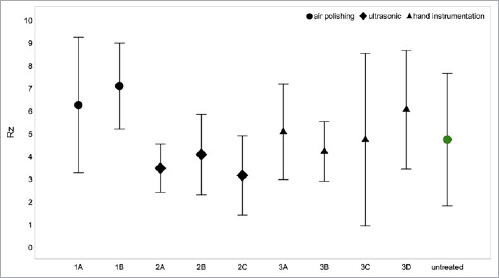
Whisker plot of mean enamel roughness (Rz) values and their standard deviations in the various groups. No statistically significant change compared to control could be found (p ≥ 0.134).

**Fig 4:**
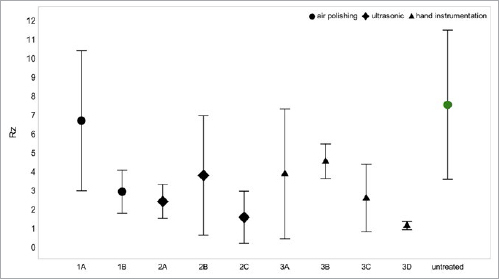
Whisker plot of mean cementum roughness (Rz) values and their standard deviations in the various groups. All treatment groups showed statistically significant alterations of the surface roughness (p ≤ 0.017) except air polishing only (1A) (p = 0.999).

The roughness averages (Ra) and maximum substance loss values measured in all treatment groups as well as the mean roughness depths measured in all treatment groups and on the untreated teeth are listed in Tables 2 and 3 and illustrated in Figs 3 and 4. Figure 5 depicts ultrastructural images of enamel and cementum for all groups, supplemented by profile charts for untreated enamel and cementum.

**Fig 5a:**
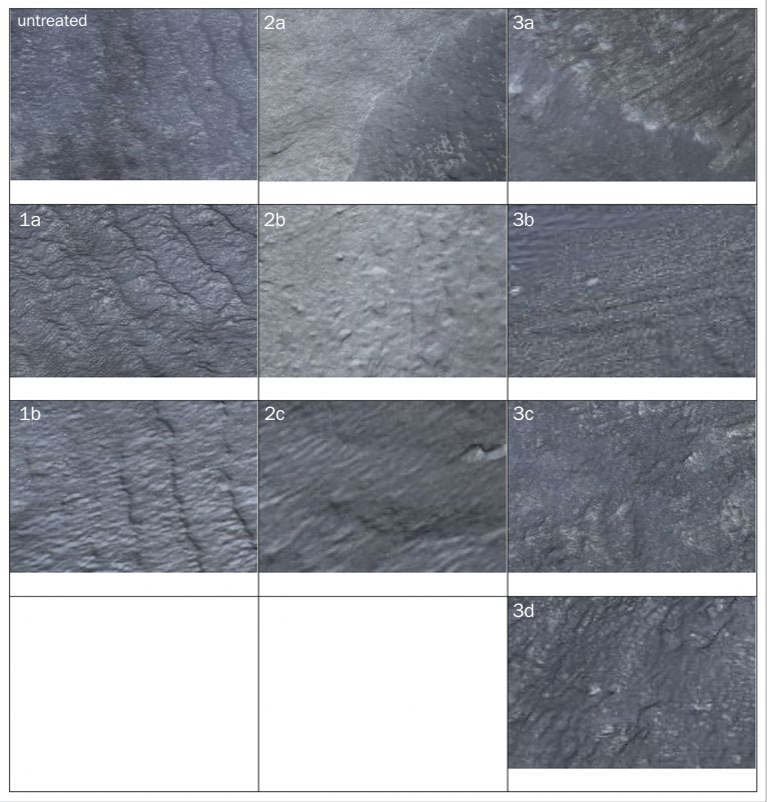
Enamel at 20X magnification: (1a) air polishing only or (1b) with rubber-cup polishing; (2a) ultrasonic scaling only or (2b) with air polishing or (2c) with rubber-cup polishing; (3a) hand scaling only or (3b) with air polishing or (3c) with rubber-cup polishing or (3d) with both.

**Fig 5b:**
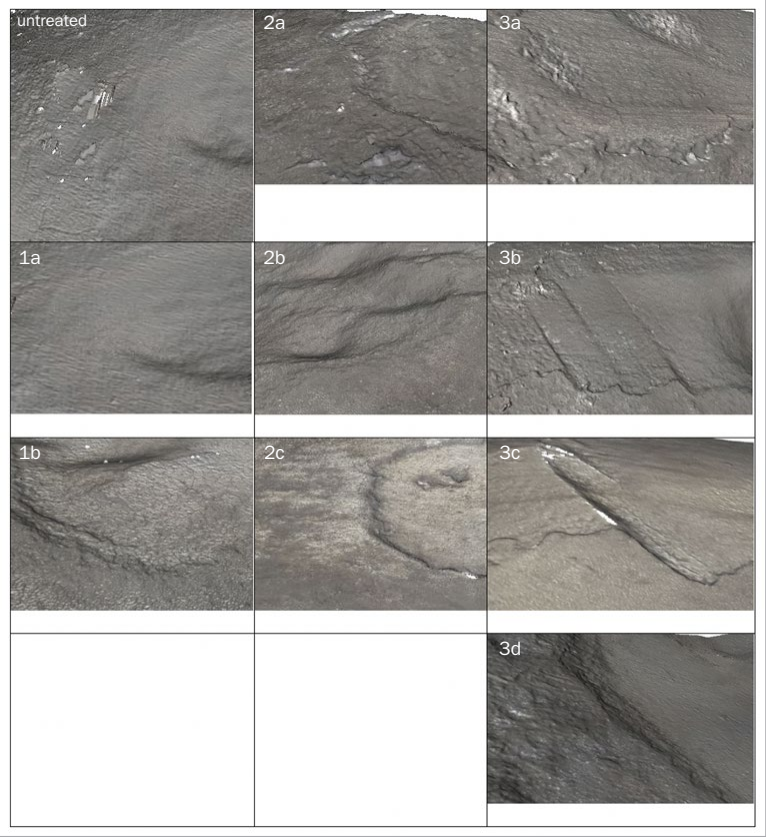
Cementum at 20X magnification: (1a) air polishing only or (1b) with rubber-cup polishing; (2a) ultrasonic scaling only or (2b) with air polishing or (2c) with rubber-cup polishing; (3a) hand scaling only or (3b) with air polishing or (3c) with rubber-cup polishing or (3d) with both.

**Table 2:** Overview of roughness averages (Ra) and maximum substance loss in the various treatment groups

Group	Enamel		Cementum	
	Ra	Loss	Ra	Loss
1A	1.2 µm	None	1.6 µm	≤ 20 µm
1B†	1.5 µm		0.6 µm	
2A	0.6 µm	< 3.0 µm	0.5 µm	≥ 20 µm
2B‡	0.7 µm		0.9 µm	
2C†	0.8 µm		0.3 µm	
3A	1.0 µm	< 3.0 µm	1.0 µm	≥ 40 µm
3B‡	0.9 µm		1.3 µm	
3C†	0.9 µm		0.4 µm	
3D†,‡	1.2 µm		0.6 µm	

Each value represents the mean of five surface samples. Group 1: air polishing with a nonabrasive powder; group 2: ultrasonic scaling; group 3: hand scaling. †Additional rubber-cup polishing with a paste; ‡additional air polishing with a nonabrasive powder.

**Table 3:** Overview of mean roughness depths (Rz) in the treatment groups and statistical comparison of techniques with additional polishing paste

	Enamel					Cementum				
Group	Rz	SD	Min	Max	p-value	Rz	SD	Min	Max	p-value
1A	6.22 µm	1.49	4.3	8.3	1A/1B = 0.999	6.64 µm	1.86	5.1	9.7	1A/1B < 0.001
1B†	7.06 µm	0.94	5.8	8.3		2.88 µm	0.57	2.0	3.5	
2A	3.44 µm	0.53	2.8	4.1		2.36 µm	0.45	1.8	3.0	
2B‡	4.04 µm	0.88	3.0	5.0	2B/2C = 0.999	3.74 µm	1.58	2.0	6.0	2B/2C = 0.051
2C†	3.12 µm	0.87	2.1	4.3		1.52 µm	0.69	1.0	2.5	
3A	5.04 µm	1.05	4.0	6.8		3.82 µm	1.72	2.5	6.7	
3B‡	4.18 µm	0.66	3.2	5.0	3B/3D = 0.174	4.48 µm	0.46	3.8	5.0	3B/3D = 0.001
3C†	4.70 µm	1.90	3.0	7.6		2.54 µm	0.89	1.5	3.8	
3D†,‡	6.02 µm	1.31	4.7	8.0		1.08 µm	0.11	1.0	1.2	
Untreated	4.70 µm	1.46	3.0	6.0		7.48 µm	1.98	5.7	10.0	

Each Rz value represents the mean of five surface samples. Group 1: air polishing with a nonabrasive powder; group 2: ultrasonic scaling; group 3: hand scaling; †Additional rubber-cup polishing with a paste; ‡additional air polishing with a nonabrasive powder.

### Enamel

Air polishing with a nonabrasive powder resulted in 6.2 µm (Rz) and 1.2 µm (Ra) when used alone (1A), as compared to 7.1 µm (Rz) and 1.5 µm (Ra) following additional rubber-cup polishing with a paste (1B). This difference in Rz was not statistically significant (p = 0.999). No substance loss of enamel was seen in either air-polishing subgroup (1A, 1B).

Ultrasonic scaling used alone (2A) caused roughness values of 3.4 µm (Rz) and 0.6 µm (Ra) on enamel. Additional air polishing (2B) raised these values to 4.0 µm (Rz) and 0.7 µm (Ra). Ultrasonic scaling combined with rubber-cup polishing (2C) resulted in roughness values of 3.1 µm (Rz) and 0.8 µm (Ra). No surface changes emerged between the groups 2B and 2C (p = 0.999). Maximum surface abrasion of the enamel layer was < 3 µm in all three groups involving ultrasonic scaling (2A, 2B, 2C).

Hand instrumentation used on its own (3A) caused roughness values of 5.0 µm (Rz) and 1.0 µm (Ra). Additional air polishing (3B) lowered these values to 4.2 µm (Rz) and 0.9 µm (Ra). Hand scaling combined with rubber-cup polishing (3C) yielded 4.7 µm (Rz) and 0.9 µm (Ra). The combination of all three techniques (3D) caused roughness values of 6.0 µm (Rz) and 1.2 µm (Ra). No statistically significant difference in Rz values between any of these subgroups involving hand scaling was observed (p = 0.174). Enamel loss was < 3 µm in all subgroups involving hand scaling (3A to 3D).

Untreated enamel displayed a mean maximum roughness profile (Rz) of 3.0−6.0 µm. None of the interventions produced statistically significant changes in Rz compared to untreated enamel (p ≥ 0.134).

### Cementum

Air polishing used alone (1A) resulted in mean roughness values of 6.6 µm (Rz) and 1.6 µm (Ra) on cementum. Additional rubber-cup polishing with a paste (1B) reduced these values to 2.9 µm (Rz) and 0.6 µm (Ra). Loss of root substance remained confined to a favorable range of ≤ 20 µm in both air-polishing subgroups (1A, 1B).

Ultrasonic scaling used by itself (2A) resulted in mean roughness values of 2.4 µm (Rz) and 0.5 µm (Ra). Additional air polishing (2B) raised these to 3.7 µm (Rz) and 0.9 µm (Ra). Ultrasonic scaling plus rubber-cup polishing (2C) yielded 1.5 µm (Rz) and 0.3 µm (Ra). Root substance loss was ≥ 20 µm in all subgroups involving ultrasonic scaling (2A, 2B, 2C).

Hand instrumentation used on its own (3A) yielded roughness values of 3.8 µm (Rz) and 1.0 µm (Ra). Additional air polishing (3B) resulted in 4.5 µm (Rz) and 1.3 µm (Ra). Hand scaling plus rubber-cup polishing (3C) resulted in 2.54 µm (Rz) and 0.4 µm (Ra). All three techniques combined (3D) led to roughness values of 1.08 µm (Rz) and 0.6 µm (Ra). Root substance loss was ≥ 40 µm in all hand-scaling subgroups (3A to 3D).

Untreated cementum showed a mean maximum roughness profile (Rz) of 5.7−10.0 µm. Air polishing when used by itself (1A) was the only intervention to reveal no significant changes in Rz from untreated cementum (p = 0.999). All other treatments statistically significantly altered the untreated cementum surface (p ≤ 0.017).

## Discussion

In vitro studies have previously demonstrated that air polishing with nonabrasive powders is a feasible method to remove plaque while inflicting little, if any, damage to the root surface.^18^ Compared to ultrasonic or sonic scalers, air-polishing systems of this type result in similar clinical parameters and offer greater patient comfort.^13,16^

Our results for surface roughness are consistent with previous findings.^3^ However, in contrast to the present results, a recent study with fifty incisor and premolar roots showed that hand scaling produced smoother surfaces than ultrasonic scalers.^21^ Since we used only impacted third molars with variable and thicker cementum layers than fully erupted incisors and premolars, and because hand instruments were only used for one stroke, comparability is questionable. Air polishing with a nonabrasive powder (erythritol plus chlorhexidine) caused no substance loss of enamel and – as the only technique evaluated – even no substance loss of cementum. Its higher Rz values compared to all other groups may be attributed to the valleys in the roughness profile becoming noticeably deeper due to its thorough cleaning capacity. Due to the limitations of this study (low caseload and no previous, comparable other study), the effect found remains an open question for further research.

Optical scanning for microcoordination measurement cannot distinguish between hard tissue and surface contaminants (e.g. abrasive particles from a prophylactic paste). The particularly low Rz values in the ultrasonic treatment groups might be due to a flattening of the natural peaks on enamel and cementum. Hand instrumentation also resulted in low roughness values on cementum, but was associated with considerably greater substance loss.

The present study demonstrates that all techniques investigated can achieve favourable roughness values on a small surface area (1–2 mm in diameter). Due to the in vitro setting, no consideration was given to any problems related to the clinical handling of each technique or to the access they offer to morphologically complex regions such as multiple roots. Ultrasonic and hand instruments result in a striped cleaning pattern via punctiform contacts with the tooth surface. Air polishing, in contrast, when applied properly at an angle of 45 degrees from a working distance of 2 mm, allows a spreading pattern, thus facilitating more uniform outcomes on large surfaces.

Uniform outcomes are much harder to achieve with ultrasonic or hand instruments, which can quickly create grooves and furrows. Any repeated instrumentation with these systems, in conjunction with excessive pressure^11^ and exposure times, will result in considerable substance loss. Our study shows that hand scalers would cause a mild loss of enamel substance (< 3 µm), thereby creating a smoother surface, with no additional smoothness to be gained by additional air and/or rubber-cup polishing.

While any polishing of cementum with pastes, brushes or rubber cups would, in clinical practice, remain confined to exposed tooth necks, this scenario was still interesting enough to be included in our study. It is also worth mentioning that polishing would, strictly speaking, imply abrasive removal of surface peaks. As no paste, brush or rubber cup can achieve such removal on hard surfaces like enamel or ceramics, the perceived smoothness could be a subjective, temporary feeling due to paste filling in the recesses of the surface profile.

This consideration – that smoothing with pastes does not occur abrasively but is merely due to paste left inside the profile valleys – is confirmed by comparing the profile valleys of the surfaces treated by rubber-cup polishing to those of untreated teeth. While polishing tools of this type are perfectly capable of removing biofilm, today’s type of air polishing with nonabrasive powders is a far more effective technique. Any surface roughness caused by other factors, two examples being parafunctional grinding or damage inflicted by acids, need to be corrected with abrasives (e.g. alumina-coated polishing disks) of descending grit sizes.

Air polishing exhibited the gentlest effect of the various techniques investigated here, offering adequate tissue preservation on cementum: an expected substance loss of up to 20 µm comes very close to the ideal of restrictin any substance loss to the 3−7 µm layer of endotoxin invasion. The limitation of this technique is its reach into deep periodontal pockets.

## Conclusion

Within the limitations of this in vitro study design, we observed no benefit – whether on enamel or cementum – of combining any of the presently available scaling and polishing techniques. Furthermore, we found these combinations to cause even more abrasion of dental hard tissue. An ideal single system of instrumentation covering all aspects of dental hard tissue is currently not available. Particularly on cementum, air polishing offers the highest hard-tissue conservation in this study. Yet it is important to note that, based on the current body of data, air polishing can only be recommended for use with powders that contain glycine or erythritol. Any routine use of bicarbonate or other heavily abrasive powders must be considered obsolete, given that their frequent application entails considerable amounts of hard-tissue loss.
